# Development and validation of a risk-prediction nomogram for patients with ureteral calculi associated with urosepsis: A retrospective analysis

**DOI:** 10.1371/journal.pone.0201515

**Published:** 2018-08-02

**Authors:** Ming Hu, Xintai Zhong, Xuejiang Cui, Xun Xu, Zhanying Zhang, Lixian Guan, Quanyao Feng, Yiheng Huang, Weilie Hu

**Affiliations:** 1 Department of Urology, Guangzhou School of Clinical Medicine, Southern Medical University (Guangzhou General Hospital of Guangzhou Military Region), Guangzhou, Guangdong, P.R. China; 2 Department of Urology, Affiliated Nanhai Hospital, Southern Medical University (People’s Hospital of Nanhai District), Foshan, Guangdong, P.R. China; 3 Department of Urology, Shunde Hospital, Southern Medical University, Foshan, Guangdong, P.R. China; "INSERM", FRANCE

## Abstract

**Objectives:**

To develop and validate an individualized nomogram to predict probability of patients with ureteral calculi developing into urosepsis.

**Methods:**

The clinical data of 747 patients with ureteral calculi who were admitted from June 2013 to December 2015 in Affiliated Nanhai Hospital of Southern Medical University were selected and included in the development group, while 317 ureteral calculi patients who were admitted from January 2016 to December 2016 were included in the validation group. The independent risk factors of ureteral calculi associated with urosepsis were screened using univariate and multivariate logistic regression analyses. The corresponding nomogram prediction model was drawn according to the regression coefficients. The area under the receiver operating characteristic curves and the GiViTI calibration belts were used to estimate the discrimination and calibration of the prediction model, respectively.

**Results:**

Multivariate logistic regression analysis showed that the five risk factors of gender, mean computed tomography(CT) attenuation value of hydronephrosis, functional solitary kidney, urine white blood cell(WBC) count and urine nitrite were independent risk factors of ureteral calculi associated with urosepsis. The areas under the receiver operating characteristic curve of the development group and validation group were 0.913 and 0.874 respectively, suggesting that the new prediction model had good discrimination capacity. P-values of the GiViTI calibration test of the two groups were 0.247 and 0.176 respectively, and the 95% CIs of GiViTI calibration belt in both groups did not cross the diagonal bisector line. Therefore the predicted probability of the model was consistent with the actual probability which suggested that the calibration of the prediction model in both groups were perfect and prediction model had strong concordance performance.

**Conclusion:**

The individualized prediction model for patients with ureteral calculi can facilitate improved screening and early identification of patients having higher risk of urosepsis.

## Background

Ureteral calculus is one of the most common diseases of urology. Ureteral calculus associated urosepsis is not rare in clinical practice. Such patients have acute onset, rapid progress, and dangerous symptoms [1, 2]. Some patients even have systemic inflammatory response syndrome and present unstable vital signs before admission; without timely and correct treatment, the condition of these patients will quickly deteriorate and will further develop into septic shock and even multiple organ dysfunction syndrome. It still has high morbidity and mortality rate even nowadays. Mortality from severe sepsis and septic shock were different in medical centers across regions and countries, with reported outcomes ranging from 22% to 76% from available epidemiological data [3].

In recent years, with an improved understanding of urosepsis by urologists, there has been a significant increase in the number of studies on risk factors of urinary tract stones leading to urosepsis [4, 5]. However, most studies focused on the risk factors of urosepsis following endoscopic lithotripsy [6–15]. Our department has observed among the admitted critically ill ureteral calculi patients that the number of patients presenting urosepsis before or right upon admission has increased year by year. Therefore, early identification of high-risk ureteral calculi patients with a tendency towards developing into urosepsis and the implementation of effective intervention methods can significantly reduce the complications and improve patient prognosis [1, 2, 16, 17].

The aim at this study is to provide a clue for the early identification and screening of high-risk patients with ureteral calculi developing into urosepsis by establishing a reliable and accurate risk-prediction model.

## Materials and methods

### Patient selection

We retrospectively analyzed the clinical data of 747 patients with ureteral calculi admitted to our hospital from June 2013 to December 2016, including 62 patients with urosepsis and 685 patients without urosepsis. A total of 317 patients with ureteral calculi admitted from January 2016 to December 2016 were enrolled in the validation cohort, including 29 patients with urosepsis and 288 patients without urosepsis.

The inclusion criteria were (1) imaging results, such as urinary system B ultrasound, excretory urogram, or abdominopelvic computed tomography (CT) leading to a diagnosis of ureteral calculi; (2) rapid increase in the sequential (sepsis-related) organ failure assessment score (SOFA), with a total score≥2 points[18]; and (3) complete laboratory and imaging data available.

### Ethics statement

The study was approved by ethics committee of Affiliated Nanhai Hospital of Southern Medical University. Written informed consent was obtained from the patients involved or their close relatives in this study. The research data were analyzed anonymously and personal identifiers were completely removed. The study was conducted in accordance with the principles contained in the Declaration of Helsinki and its later amendments.

### Risk factors

We collected and analyzed the following factors of the subjects: general information (gender, age, diabetes, hypertension, and previous surgery for calculi), characteristics of ureteral calculi (length of calculi size, width of calculi size, mean CT attenuation value of calculi, laterality of calculi, location of calculi, and ipsilateral renal calculi), characteristics of the affected kidney (mean CT attenuation value of hydronephrosis, degree of hydronephrosis, and functional solitary kidney), and urine test results (urine white blood cell (WBC) count and urine nitrite).

The length and width of ureteral calculi size were measured by abdominal X-ray kidney-ureter-bladder (KUB) and/or B ultrasound and/or CT. The mean CT attenuation values of ureteral calculi and degree of hydronephrosis were revealed and detected using the PACS image software of our hospital. For irregular calculus and hydronephrosis, the hounsfield unit (HU) value of the maximum annular range was taken as the mean CT attenuation value.

Degree of hydronephrosis was defined as mild, moderate and severe. Mild hydronephrosis was defined as renal pelvis dilatation without dilatation of calyces, moderate hydronephrosis was defined as dilatation of renal pelvis and calyces without parenchymal atrophy, and severe hydronephrosis was defined as gross dilatation of renal pelvis and calyces with parenchymal atrophy[19].

Functional solitary kidney was defined as either a history of contralateral nephrectomy or by confirmation of poor split renal function with radionuclide imaging method.

### Statistical analysis

Our analysis showed that the measurement data in this study were not normally distributed. Therefore, the measurement data were expressed as medians (quartiles), and the count data were expressed as frequencies (percentages). The measurement data were analyzed using the Mann-Whitney U test, and the count data were analyzed using the χ^2^ test. Risk factor analysis was performed using univariate and multivariate logistic regression analyses. Variables showing statistical significance of the univariate analysis were included in the multivariate logistic regression analysis, and the forward stepwise method was used to select the variables that were eventually included in the model.

Based on the regression coefficients of independent variables, we established the individualized nomogram prediction model of ureteral calculi associated with urosepsis [20, 21]. The prediction model was evaluated in terms of discrimination and calibration. The discrimination of prediction model refers to its ability to distinguish between patients with ureteral calculi developing into urosepsis from those without into urosepsis. A dichotomized outcome discrimination is most often assessed by calculating the area under the curve (AUC) of the receiver operating characteristic (ROC) curve[22]. The AUC value is between 0.5 and 1.0. The closer the AUC value is to 1, the better discrimination capacity the prediction model has. Generally, a prediction model that performs with an AUC of 0.5–0.75 is considered acceptable, and AUC>0.75 indicates the model shows excellent discrimination [23].

The calibration of prediction model refers to the concordance between the predicted and observed probabilities. A novel statistical test, the GiViTI calibration belt was introduced into the development group and validation group to investigate the goodness of fit of the prediction model[24]. The GiViTI calibration belt was designed to disclose the relationship between predicted probabilities and observed probabilities by fitting a polynomial logistic function. And it also calculates the 80% CI (light gray area) and 95% CI (dark gray area) in the calibration belt plot, respectively. When the 95% CI does not cross the bisector, statistically significant deviation from the bisector vector occurs. Wider confidence intervals are considered as a higher degree of uncertainty, for tiny proportion of patients is at the specific risk interval[25]. Small P-value(P<0.05) provides evidence that the prediction model’s calibration is not perfect. Large P-value of GiViTI calibration test suggests that there is not strong evidence of model’s lack of fit.

Statistical analysis was performed using SPSS software (ver 20.0, USA), MedcCalc software (ver 18.2.1, Belgium) and R software (ver 3.4.0, USA). The ROC curve was plotted using MedCalc software, and the GiViTI calibration belt was drawn by RMS-package. Two-tailed analysis with P<0.05 indicated that the difference was statistically significant.

## Results

### Patient demographics

In this study, a total of 1064 patients were enrolled, including 747 in the development group and 317 in the validation group. There were 591 males and 473 females (aged 53 years(43–63 years)). A total of 106 patients had diabetes, 272 cases had hypertension, and 241 cases had previous surgery for calculi. The average length of calculi size was 10(7 ~ 14) mm, the average width of calculi size was 6(5 ~ 8) mm, and the mean CT attenuation value of calculi was 399.0(237.5 ~ 649.75) HU. There were 545 cases with calculi located on the left side, and there were 519 cases with calculi located on the right side. There were 544 cases with upper ureteral calculi, 156 cases with middle ureteral calculi, and 364 cases with lower ureteral calculi. A total of 451 cases had ipsilateral renal calculi. The mean CT attenuation value of hydronephrosis was 3(1 ~ 7) HU, and the numbers of cases with functional solitary kidney, mild hydronephrosis, moderate hydronephrosis, severe hydronephrosis, positive urine WBC count and positive urine nitrite are listed below (Table 1).

**Table 1:** Baseline characteristics of the development group and validation group.

	Development group(n = 747)	Validation group (n = 317)	Z/χ^2^	P Value
Gender (%)			0.912	0.346
Male	422(56.5)	169(53.3)		
Female	325(43.5)	148(46.7)		
Age, year	53(43~63)	52(43~64)	0.298	0.765
Diabetes (%)				
No	677(90.6)	281(88.6)	0.978	0.323
Yes	70(9.4)	36(11.4)		
Hypertension (%)				
No	567(75.9)	225(71.0)	2.838	0.105
Yes	180(24.1)	92(29.0)		
Previous surgery of calculi (%)			0.591	0.472
No	573(76.7)	250(78.9)		
Yes	174(23.3)	67(21.1)		
Functional solitary kidney (%)				
No	691(92.5)	292(92.1)	0.048	0.802
Yes	56(7.5)	25(7.9)		
Maximum diameter of calculi, mm	10(7~14)	10(6–14)	0.092	0.927
Minimum diameter of calculi, mm	6(5~8)	6(4~8)	1.002	0.316
Mean CT attenuation value of calculi, Hu			0.715	0.699
<500	441(59.0)	195(61.5)		
500~1000	251(33.6)	102(32.2)		
>1000	55(7.4)	20(6.3)		
Laterality of calculi (%)			0.569	0.461
Left	377(50.5)	168(53.0)		
Right	370(49.5)	149(47.0)		
Location of calculi (%)			1.850	0.397
Upper	382(51.2)	162(51.1)		
Middle	116(15.5)	40(12.6)		
Lower	249(33.3)	115(36.3)		
Ipsilateral renal calculi (%)			2.490	0.119
No	442(59.2)	171(53.9)		
Yes	305(40.8)	146(46.1)		
Mean CT attenuation value of hydronephrosis, Hu			2.127	0.345
<8	573(76.7)	231(72.9)		
8~16	132(17.7)	68(21.4)		
>16	42(5.6)	18(5.7)		
Degree of hydronephrosis (%)			3.769	0.152
Mild	454(60.8)	173(54.6)		
Moderate	164(22.0)	84(26.5)		
Severe	129(17.2)	60(18.9)		
Urine WBC count (%)			0.761	0.859
No	384(51.4)	155(48.9)		
Weakly positive	184(24.6)	81(25.6)		
Moderately positive	100(13.4)	43(13.6)		
Strongly positive	79(10.6)	38(12.0)		
Urine nitrite (%)			0.191	0.669
No	705(94.4)	297(93.7)		
Yes	42(5.6)	20(6.3)		

CT, computer tomography; WBC, white blood cells.

Comparison of the baseline data indicated that the development and validation groups showed no significant differences in the general situation of patients, characters of ureteral calculi, ipsilateral kidney characteristics, and other indicators.

### Nomogram development

Univariate analysis of the development group showed that the statistically significant risk factors were gender, age, diabetes mellitus, hypertension, previous surgery for calculi, functional solitary kidney, length of calculi size, width of calculi size, mean CT attenuation value of calculi, location of calculi, associated ipsilateral renal calculi, mean CT attenuation value of hydronephrosis, urine WBC count and urine nitrite (P<0.05), whereas the laterality of calculi and degree of hydronephrosis were not related to urosepsis.

Statistically significant variables screened from the univariate analysis were included in the non-conditional binary multivariate logistic regression. The five factors of gender, mean CT attenuation value of hydronephrosis, functional solitary kidney, urine WBC count and urine nitrite were independent risk factors of ureteral calculi associated with urosepsis (Table 2) (P<0.05). We conducted collinearity diagnostics for the above independent risk factors, and the variance inflation factors (VIFs) were 1.049, 1.012, 1.027, 1.203 and 1.128 respectively, suggesting that there was no multiple collinearity among the five independent risk factors.

**Table 2:** Univariate and multivariate logistic regression models in the development group.

	Univariate analysis		Multivariate analysis	
	OR (95%CI)	P value	OR (95%CI)	P value
Gender	4.59(2.52~8.37)	<0.001	4.54(2.14~9.62)	<0.001
Age, years	1.05(1.03~1.08)	<0.001	NA	
Diabetes	2.25(1.11~4.45)	0.024	NA	
Hypertension	2.14(1.25~3.68)	0.006	NA	
Functional solitary kidney	4.47(2.28~8.75)	<0.001	3.02(1.28~7.14)	0.012
Previous surgery of calculi	2.43(1.42~4.17)	0.001	NA	
Length of calculi size(mm)	1.07(1.03~1.11)	<0.001	NA	
Width of calculi size(mm)	1.11(1.04~1.18)	0.002	NA	
Mean CT attenuation value of calculi (HU)	1.00(1.00~1.00)	0.001	NA	
Laterality of calculi	1.25(0.74~2.10)	0.408	NA	
Location of calculi	0.69(0.50~0.95)	0.021	NA	
Ipsilateral renal calculi	3.37(1.94~5.87)	<0.001	NA	
Mean CT attenuation value of hydronephrosis (HU)	2.61(1.83~3.73)	<0.001	3.17(2.00~5.04)	<0.001
Degree of hydronephrosis	1.22(0.89~1.68)	0.222	NA	
Urine WBC count	3.69(2.78~4.90)	<0.001	2.94(2.14~4.03)	<0.001
Urine nitrite	10.77(5.55~20.90)	<0.001	4.71(2.08~10.69)	<0.001

OR, odds ratio; CI, confidence interval; NA, not available; HU, Hounsfield unit.

**Fig 1:**
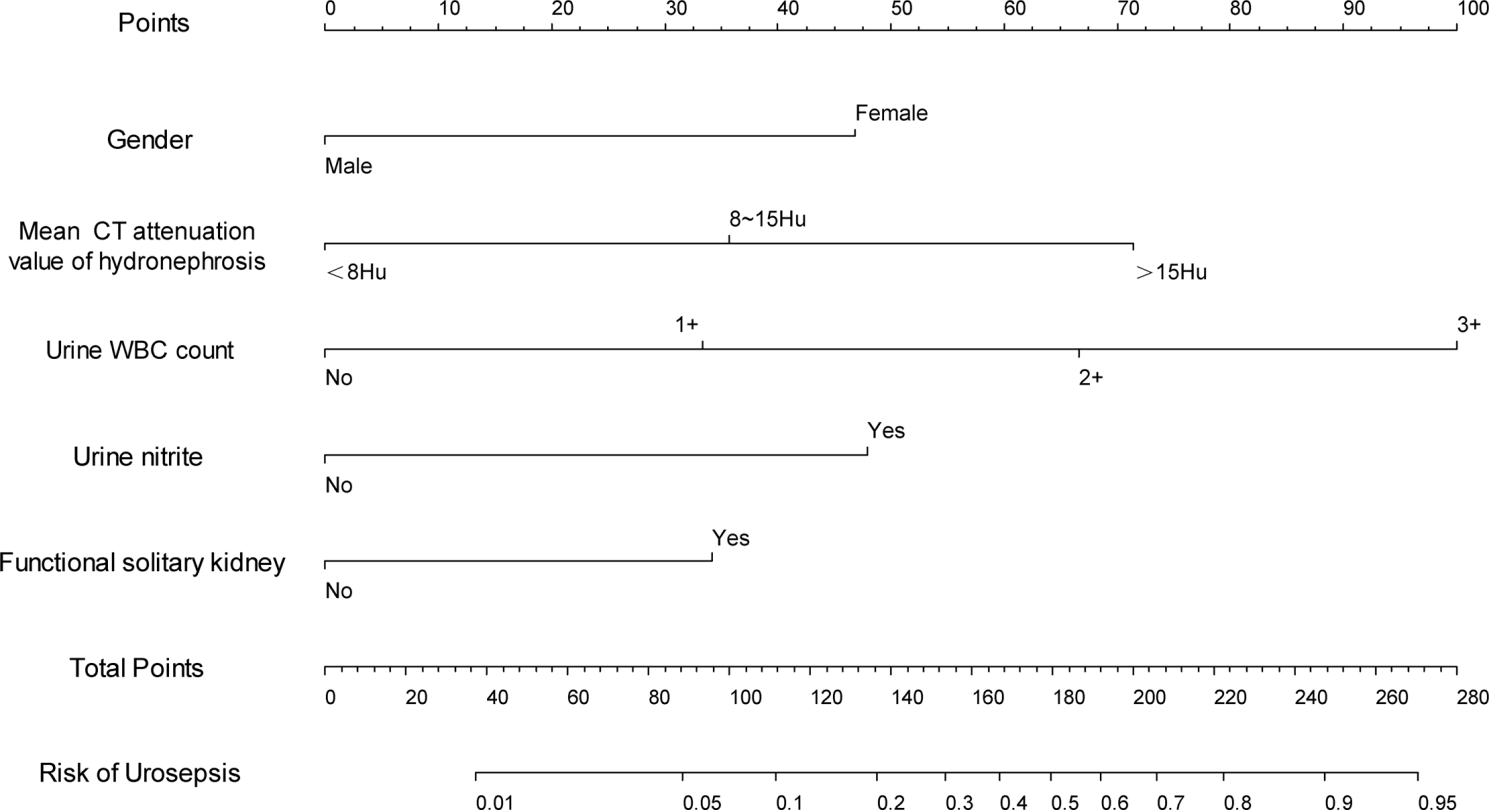
Nomogram to predict the probability of urosepsis in the patient with ureteral calculi.

Based on the logistic multivariate regression analysis, the five independent risk factors were included in the prediction model. We then establish an individualized nomogram prediction model of ureteral calculi associated with urosepsis (Fig 1). The application of the nomogram is as follows: based on the nomogram, we can obtain the points corresponding to each prediction indicator, the sum of the points is recorded as the total score, and the predicted risk corresponding to the total score is the probability of ureteral calculi associated with urosepsis.

**Fig 2:**
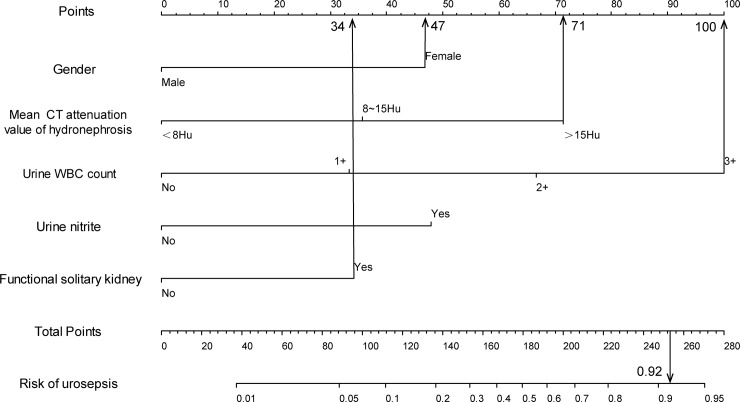
Example prediction nomogram for risk of urosepsis in a patient with ureteral calculi.

For example, an elderly female patient with ureteral calculi (47 points) had a mean CT attenuation value of ipsilateral hydronephrosis at 16 HU (71 points), an urine WBC count +++ (100 points), negative urine nitrite (0 points), and unilateral renal atrophy in CT (34 points). The cumulative score of the various prediction indicators was 47 +71 +100 +0 +34 = 252, and the corresponding predicted risk of urosepsis was 0.92 (92%) (Fig 2). According to the predicted probability above, this patient has high-risk of urosepsis.

### Nomogram validation

The validation of the model was based on discrimination and calibration. We drew the ROC curves of predicted probability and calculated the AUC values in the development and validation group [22]. The ROC curve was used to compare the AUC values of the five independent risk factors of the nomogram and multivariate analysis (Table 3), and the differences were statistically significant (P<0.05).

**Table 3:** The AUCs of the ROC curves for the nomogram and variables from the logistic regression model in the development group and validation group.

	Development group			Validation group		
	AUC	95%CI	P value	AUC	95%CI	P value
Nomogram variable	0.914	0.88~0.95	<0.001	0.874	0.80~0.95	<0.001
Gender	0.676	0.61~0.74	<0.001	NA		
Functional solitary kidney	0.582	0.50~0.66	0.032	NA		
Mean CT attenuation value of hydronephrosis	0.647	0.57~0.73	<0.001	NA		
Urine WBC count	0.863	0.82~0.90	<0.001	NA		
Urine nitrite	0.634	0.55~0.72	<0.001	NA		

ROC, receiver operating characteristic; AUC, area under the curve; CI, confidence interval.

**Fig 3:**
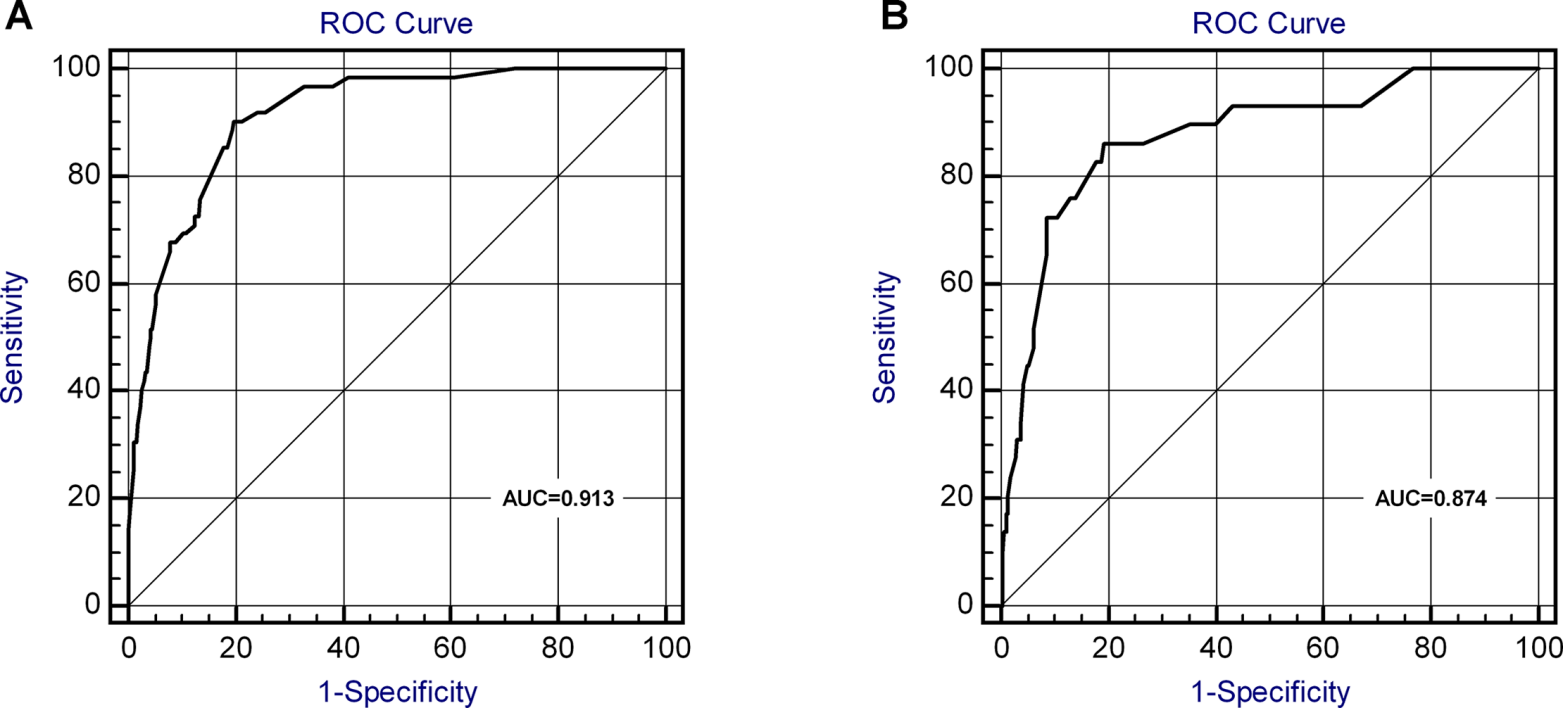
ROC curves for validating the discrimination power of the nomogram. (A) Development group. (B) Validation group. (AUC = 0.914 vs. 0.874).

The AUC values for urosepsis risk of the development group and validation group were 0.914 and 0.874 (Fig 3) respectively, suggesting that the nomogram prediction model has an excellent discrimination.

**Fig 4:**
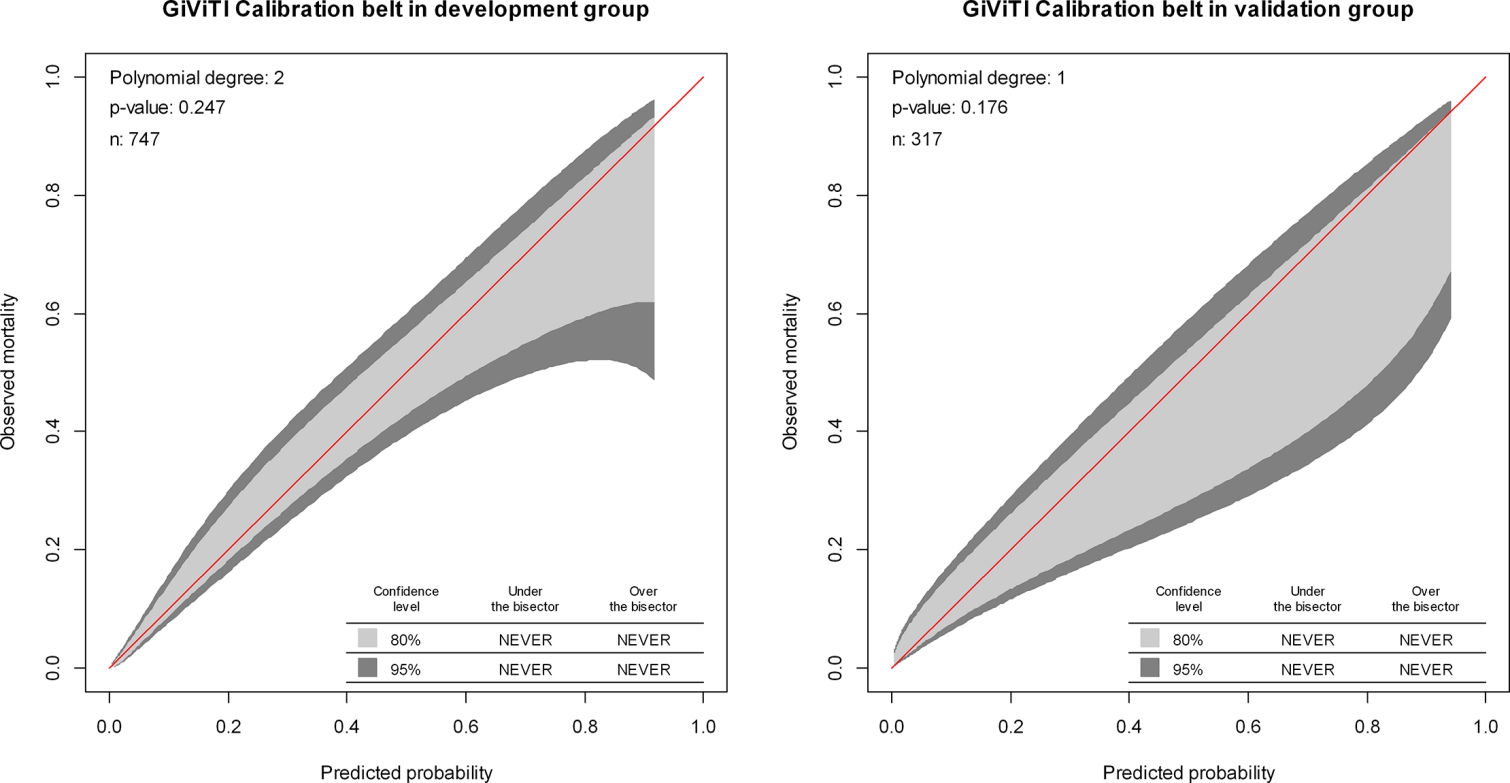
Calibration plots of the nomogram for the probability of urosepsis patients with ureteral calculi in the development group and validation group.

The 95% CIs of GiViTI calibration belt in both development and validation groups did not cross the diagonal bisector line, and the P-value in GiVITI calibration test of the two groups were 0.247 and 0.176 respectively (Fig 4). Therefore the predicted probability of the model was consistent with the actual probability which suggested that prediction model had strong concordance performance, and the calibration of the prediction model in the both groups were perfect [25].

## Discussion

Urosepsis is a form of sepsis caused by urinary tract infection [1, 17], accounting for approximately 9–31% of all cases of sepsis [3]. In recent years, with improved understanding of urosepsis and the appearance of a large number of clinical studies, rich collections of clinical evidence for the standardized treatment of urosepsis have become available. Consequently, the mortality of urosepsis has decreased over the years [1, 2, 17]. However, the incidence of urosepsis in the world still shows an increasing trend year by year [2]. Frequently underlying risk factors of urosepsis are urinary tract obstruction, such as calculi, prostate hyperplasia, oncothlipsis obstruction urethral stricture and congenital anomalies. The most common cause of urosepsis in urinary tract obstruction as we know is ureteral calculi [17]. Endoscopic surgeries in urinary tract and transrectal prostate biopsies also results in urosepsis.

Previously, it was believed that sepsis is a systemic response to infection. And the signs and symptoms of systemic inflammatory response syndrome (SIRS), which were initially considered to be compulsive diagnosis of sepsis [26, 27], are viewed as alerting symptoms nowadays [28]. However, in clinical practice, the diagnostic specificity and sensitivity based on the diagnostic criteria of systemic inflammatory response syndrome is not sufficient and cannot truly reflect this abnormal life-threatening body reaction. Kaukonen et al. [29] found that approximately 1/8 of patients failed to meet the SIRS diagnostic criteria even in the presence of systemic infection and multiple organ dysfunction. Recently, the definitions of sepsis were updated and published by ‘The Third International Consensus Definitions for Sepsis and Septic Shock’ (sepsis 3). The European Society of Intensive Care Medicine and the Society of Critical Care Medicine revised the diagnostic criteria for sepsis in January 2014 and emphasized that host response disorders and fatal organ dysfunction are important differences between sepsis and infection. According to the degree of organ dysfunction, the new scoring system was composed of scores from six organ systems (respiratory, cardiovascular, hepatic, coagulation, renal and neurological) ranged from 0 to 4. They recommended that for patients with unknown basic organ dysfunction, the baseline SOFA score should be set to 0, and a rapid increase in the SOFA score after infection to no less than 2 should be used as the criterion for the clinical determination and screening of sepsis. A SOFA score of 2 points or more for a patient is associated with an in-hospital mortality greater than 10%. Sepsis is essentially a life-threatening organ dysfunction due to misregulated host responses to infection, i.e., when the body's response to infection damages its own tissues and organs; when this condition becomes life-threatening, it can be called sepsis [2, 18]. The European Association of Urology (EAU) also agrees with the SOFA scoring system and uses it as a new diagnostic criterion for urosepsis in its diagnosis and treatment guidelines in 2017.

Gender is an independent risk factor of urosepsis in patients with ureteral calculi, i.e., the risk of urosepsis in female patients with ureteral calculi is approximately 4.5 times that of male patients. Some other published literature had the same viewpoint [7, 30]. However, a meta-analysis written by Peach BC et al. [5] on the risk factors of urosepsis in the elderly showed that a number of studies differed from whether age, gender, race, complications, and pathogenic microbial species were risk factors for urosepsis. In animal experiments, Kawasaki et al. [31] found that after shock, trauma, or stimulation of sepsis factors, ovariectomized animals or old animals showed significantly inhibited immune function and organ response capacity. Kumar et al. [7] suggested that the lack of hygiene in the perineum, postmenopausal estrogen deficiency, atrophic vaginitis, cystocele, and the use of vaginal pessary might be the causes of the tendency of elderly women to develop SIRS and urine culture positivity.

The CT attenuation value is the value corresponding to the X-ray attenuation coefficient of various organs in CT images and can be used to determine the density of local tissues or organs of the human body. A higher mean CT value of renal hydronephrosis suggests denser and more viscous liquid accumulation in hydronephrosis, and therefore, a greater possibility of pyonephrosis. Pyonephrosis usually indicates the presence of urinary tract obstruction. Long-term chronic obstruction causes severe local infection, and upon the presence of predisposing factors, the risk of urosepsis naturally increases. Yuruk et al. [32] showed that the CT values of pus in pyonephrosis patients were significantly higher than those of patients with hydronephrosis; the difference was statistically significant. Because pus often contains infectious substances, cell debris, and large numbers of micro-organisms, the CT value of the pus is higher than hydronephrosis, and application of CT values based on CT thin-layer scanning to identify hydronephrosis and pyonephrosis yields satisfactory results [33]. In clinical practice, a significant increase in CT attenuation value of hydronephrosis should raise the possibility of pyonephrosis, and an inadequately prepared endoscopic procedure can easily induce urosepsis.

This study also showed that the two urine-related detection indicators were closely related to urosepsis and that there was no collinear relationship between urinary WBC count and urine nitrite. Consistent with previous studies, the above results in our study indicated that the two indicators were independent risk factors for ureteral calculi associated with urosepsis [6, 8, 9, 11, 14]. But some other scholars still hold different opinions [15, 34]. They thought that positive mid-stream urine culture or positive stone culture was associated with significantly associated with post-operative urosepsis respectively. In our opinion, urine culture has certain hysteresis characteristic. Urine culture result usually takes 2–3 days in most hospitals. Our prediction model aimed to improve the early identification and screening of high-risk patients of urosepsis. So we gave up to choose urine culture into the candidate risk factors.

If both of the two indicators are positive, and the urine WBC count is +++, the total score of the nomogram is 148(100+48), suggesting that positive urine infection indicators can increase the urosepsis risk of patients with ureteral calculi by 53% (148/280). Such patients often have more severe urinary tract infections, and if the calculi suddenly move downward or if the patients receive minimally invasive endoscopic surgery, there can be a sudden increase in renal pelvic pressure. Calculi and iatrogenic procedures may cause mechanical damages to ureteral mucosa and nourishing veins, mucosal barrier function is destroyed, and turbid urine suddenly enters the blood via reflux mechanisms, such as renal pelvis-lymphatic vessels, renal pelvis-vein, renal pelvis-renal sinus, and renal pelvis-renal tubules [35]. As a result, pathogens or endotoxin from urine or calculi can also be released in large quantities and then invade the circulatory system to stimulate the body to produce endogenous inflammatory mediators, which further stimulates the body to produce SIRS. And then a burst of second messenger molecules results in several different stages of the septic process, from hyperactivity to immune suppression [17].

In clinical practice, we should have a systematic and comprehensive understanding of the urine test indicators and cannot just stay in the level of the diagnosis of general urinary tract infections. We should consider whether patients have pathogenic factors leading to complex urinary tract infections, whether patients have a risk of urosepsis and other serious complications, and how to avoid recurrence of urinary tract infection and reduce the recurrence rate.

In addition, we cannot ignore patients suffered from ureteral calculi with negative urine examination results [6]. Some patients have long-term chronic obstructions with infection and develop tolerance toward pain. In such patients, stones containing bacteria could completely obstruct upstream urine, similar to ‘autonephrectomy’, which leads to a false-negative urine analysis results, even in the presence of pyonephrosis, and thus covers up the disease [34, 36]. In this situation, the advantages of this nomogram prediction model are beneficial, and the risk of urosepsis in these patients can be evaluated by the other observation indexes of the prediction model.

Patients with functional solitary kidney often have varying degrees of renal insufficiency but have not yet reached the hemodialysis indications. Once ureteral calculi cause obstruction, these patients would show rapid deterioration of renal function within a short time frame and would present with acute renal failure [37, 38]. If this condition is combined with infection, it becomes more dangerous. This study found that in the presence of ureteral calculi, the risk of urosepsis in functional solitary kidney patients was about 3 times that of the normal person. Therefore, if functional solitary kidney patients show symptoms of renal colic or fever, renal function and urosepsis risk should be assessed as soon as possible to facilitate early intervention and to avoid rapid deterioration and disease progression [39].

The nomogram we developed can be applied for both outpatients and inpatients with ureteral calculi, not only in those inpatients who are ready to undergo surgery. The nomogram is a beneficial supplementary tool for clinical work, and it also makes patients more aggressive in the decision-making process with regard to their diagnosis and treatment.

There are also shortcomings in this study. (1) It is a retrospective study, which cannot avoid selection bias. However, we strictly set the inclusion criteria and collected relatively adequate clinical samples so that the case and control groups of patients can truly reflect the actual condition of disease occurrence. (2) The data for the prediction model were derived from a single center. Although we used patient samples from different periods to validate the model, we still need evidence from other centers for validation. Therefore, in the follow-up research work, we will persuade other medical centers to join this research project and will provide the appropriate clinical data to conduct a more in-depth assessment and validation of the prediction model.

## Conclusion

We established an individualized nomogram prediction model for ureteral calculi developing into urosepsis. Through this prediction model, we can accurately predict the risk of urosepsis in patients with ureteral calculi, which helps to improve the early identification and screening of such high-risk patients.

## Supporting information

S1 File Development group data. The raw dataset of development group.
(SAV)

S2 File Validation group data. The raw dataset of validation group.
(SAV)
